# The Rationalization of Surgical Trays in Staged Bilateral Lower Limb Arthroplasty: A 10-Year Cohort Study

**DOI:** 10.7759/cureus.69665

**Published:** 2024-09-18

**Authors:** Joseph Boktor, Rishi Trivedi, Awf A Alshahwani, Vinay Joseph, Ahmed Ashry, Peter Lewis

**Affiliations:** 1 Trauma and Orthopaedics, Cardiff University Hospital, Cardiff, GBR; 2 Trauma and Orthopaedics, Leicester Royal Infirmary, Leicester, GBR; 3 Trauma and Orthopaedics, Leicester University Hospital, Leicester, GBR; 4 Trauma and Orthopaedics, Glangwili General Hospital, Carmarthen, GBR; 5 Trauma and Orthopaedics, Prince Charles Hospital, Merthyr Tydfil, GBR

**Keywords:** cost reduction, orthopedic surgical tray rationalization, retrospective cohort, staged bilateral total hip arthroplasty, staged bilateral total knee arthroplasty

## Abstract

Background

Surgical tray rationalization involves minimizing surgical equipment to reduce operating theater expenses. This study aims to assess whether rationalization of surgical trays is possible in a staged bilateral total hip or total knee replacement by utilizing the first surgical tray as a reference.

Methodology

A retrospective analysis was conducted of a consecutive cohort of staged bilateral lower limb arthroplasties from August 2009 to February 2020. The staged procedures were performed by the same surgeon using the same technique and the same implant system between the sides.

Results

A total of 442 out of 511 consecutive staged lower limb arthroplasties were included. For bilateral total knee replacements (BTKRs), 146 joints were operated on in 73 patients. The mean interval between sides was 28 months. Overall, 72/73 (98.6%) patients had both tibial and femoral components that were within one size of the first side operated on. For bilateral total hip replacements (BTHRs), 296 joints were operated on in 148 patients. The time interval between sides was 24 months. Overall, 140/148 (94.6%) patients had an acetabular cup size that was within a one-size difference between the first and second-side surgery. Regarding differences in femoral stem sizes, 130/148 (87.8%) had an implant that was again within a one-size difference between the first and second-side surgery. Our results demonstrated that the rationalization of surgical trays can be adopted for patients who have implants that are within one size of the first side operated on in both BTKRs and BTHRs. This has the potential to reduce costs by £159.81 and £151.26 per case, respectively.

Conclusions

This cohort study confirms implant sizes used for first-side surgery are a reliable predictor for those used in second-side surgery in staged bilateral lower limb arthroplasty. Used in conjunction with preoperative templating, the surgical team can confidently rationalize surgical trays, thereby improving theater efficiency and decreasing sterilization costs.

## Introduction

There has been a global increase in healthcare service demand and an associated increase in the funding required. An overloaded healthcare system can expose patients to risks and can compromise the quality of care [1,2]. Hip and knee joint replacements are among the most in-demand of all elective surgical operations performed within the National Health Service (NHS) [3,4]. In 2019, 109,629 total hip replacements (THRs) and 115,514 total knee replacements (TKRs) were undertaken and added to the National Joint Registry (NJR) [5,6]. The COVID-19 pandemic in 2020 resulted in a further global strain on healthcare, leading to overburdening of elective orthopedic waiting lists, especially joint replacement surgery [7]. It is estimated that there are currently 140,000 patients on the waiting list for elective orthopedic surgery in the United Kingdom [7]. With the average cost recognized to be £4,000 per operation, this equates to a total of 2.6 billion pounds to clear the present waiting list [8,9].

It is important to explore every possible avenue to deliver cost-effective services. Surgical tray rationalization (STR) is the principle of a systematic reduction in the number of surgical instruments and/or trays to perform specific procedures. This is without compromising patient safety and while reducing costs in the sterilization and assembly of the trays [2]. In the United Kingdom, 98.7% of TKRs and 99.5% of THRs are performed as staged procedures [5,6].

This study reviews the possibility of rationalizing surgical trays in a staged bilateral total hip or total knee replacement by utilizing the first surgical tray as a reference. The first tray can act as a reference for choosing and minimizing implant preparation equipment. In turn, it is possible to calculate the possible reduction in cost on an annual basis per surgeon as well as the potential reduction in costs across the NHS.

## Materials and methods

We conducted a retrospective analysis of a consecutive cohort of staged bilateral lower limb arthroplasties from August 2009 to February 2020.

Inclusion criteria

All staged bilateral lower limb arthroplasties performed using the same approach (standard posterior hip approach/standard medial para-patellar knee approach) and technique were considered for inclusion. All procedures were undertaken by the same lead surgeon using matching implant brands and types between sides. The minimal follow-up was one year. The indications for surgery were osteoarthritis (OA), either primary or secondary (e.g., Perthes disease, adult dysplasia, or avascular necrosis).

Exclusion criteria

We excluded unilateral lower limb arthroplasties or if the second side was operated on by a different surgeon. In addition, we excluded cases if one side had a different implant type and/or brand, if one side was revised, if a cemented hip or a special implant was used, and if one or both sides had a different indication other than OA for surgery or unilateral pathology such as post-traumatic arthritis.

Data extraction

All data were collected from a single surgeon’s documentation and clinic letters. Postoperative complications and long-term follow-up were recorded. Ethical approval was not sought due to the retrospective nature of the study and the anonymization of patient identifiers throughout. However, approval for the study was sought after and granted upon discussion with the research and development department of the health board.

Research questions

We had three principle research questions. The first was whether the size of the implant used in first-side surgery can predict the size of the implant used in second-side surgery in staged bilateral lower limb arthroplasty. Second, whether the STR principle can be applied in staged bilateral lower limb arthroplasty. If yes, what is the average reduction in costs to the NHS annually?

Outcomes

The primary outcome was to determine the difference in implant sizes between first and second-side surgery in the same patient. Secondary outcomes included the interval between first and second-side surgery and patient demographics. Additionally, an analysis of the difference in implant sizes and meaningful improvement in patient-reported outcome measures (PROMs) following second-side surgery was performed.

Statistical analysis

The anonymized data set was analyzed using SPSS Statistics software version 27 (IBM Corp., Armonk, NY, USA). Continuous variables were analyzed using a paired t-test or independent-sample t-test where indicated, and the chi-square test was used for categorical variables. Where a comparison of three or more groups was required, a one-way analysis of variance was used, with Tukey post hoc analysis for the differences between individual groups.

## Results

Total knee arthroplasty

**Figure 1:**
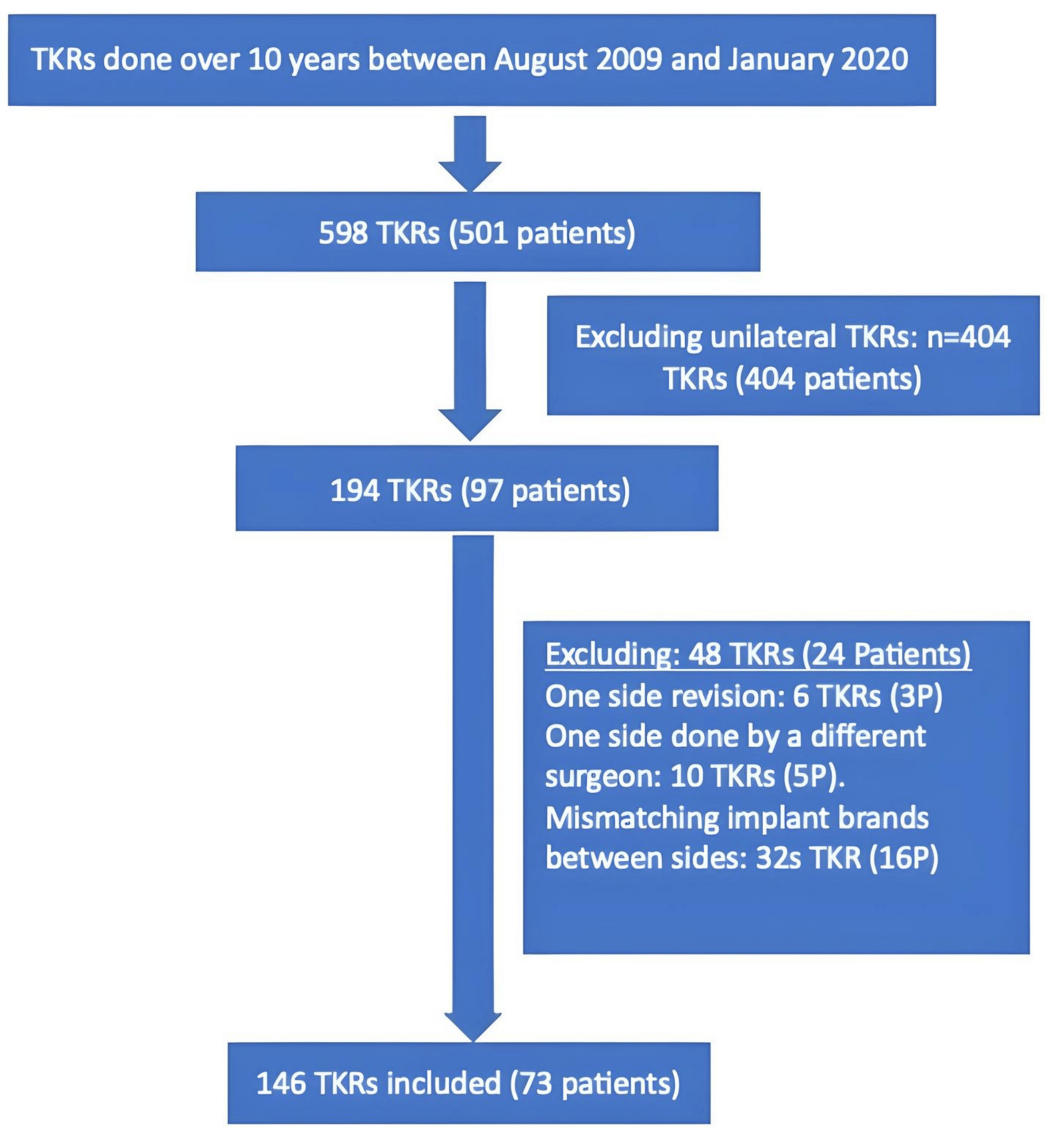
All total knee replacements (TKRs) performed by the same surgeon over the defined study period. The selection of the study group and reasons for exclusion are presented.

For bilateral total knee replacements (BTKRs), 146 joints were operated on in 73 patients (Figure 1). Most patients (54/73) were aged over 60 years, and 42/73 were female (Table 1). The mean body mass index (BMI) of the patient at first and second-side surgery was 35.51 and 35.67, respectively. All patients had primary OA in both knees. The mean interval between first and second-side surgery was 28 months (range = 3-108 months). Detailed implant sizes were available in all cases (73/73). Overall, 72/73 patients received a sigma PFC (DePuy Synthes) on both sides, and a single patient received a Triathalon (Stryker) implant. Further, 130/146 TKRs performed were cruciate retaining. Only 16/146 were posterior stabilizing TKRs. None of the TKRs needed patellar resurfacing.

**Table 1:** Patient demographics including age, sex, and BMI (TKR). BMI: body mass index; TKR: total knee replacement

Variables		n = 73
Sex	Male	31 (42.47%)
	Female	42 (57.53%)
Age at the time of the first side	40–60	19 (26.03%)
	60–80	48 (65.75%)
	>80	6 (8.33%)
First side laterality	Right	41 (56.16%)
	Left	32 (43.84%)
BMI at the first side	<25	5 (6.85%)
	25–30	9 (12.33%)
	30–40	35 (47.9%)
	>40	17 (23.29%)
	N/A	7 (9.59%)

**Figure 2:**
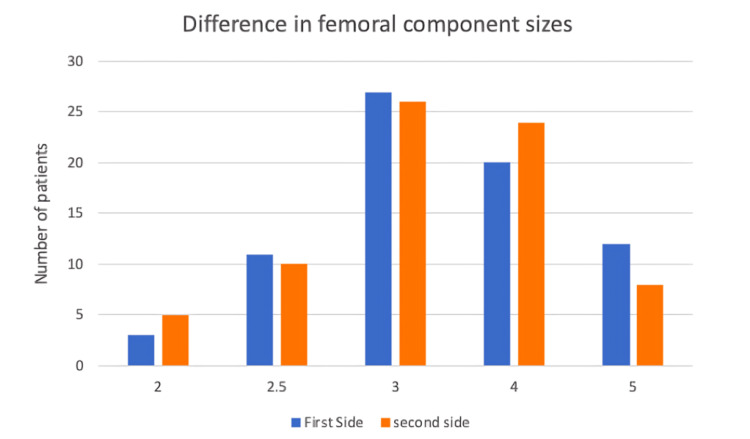
Femoral component sizes used in the cohort.

**Figure 3:**
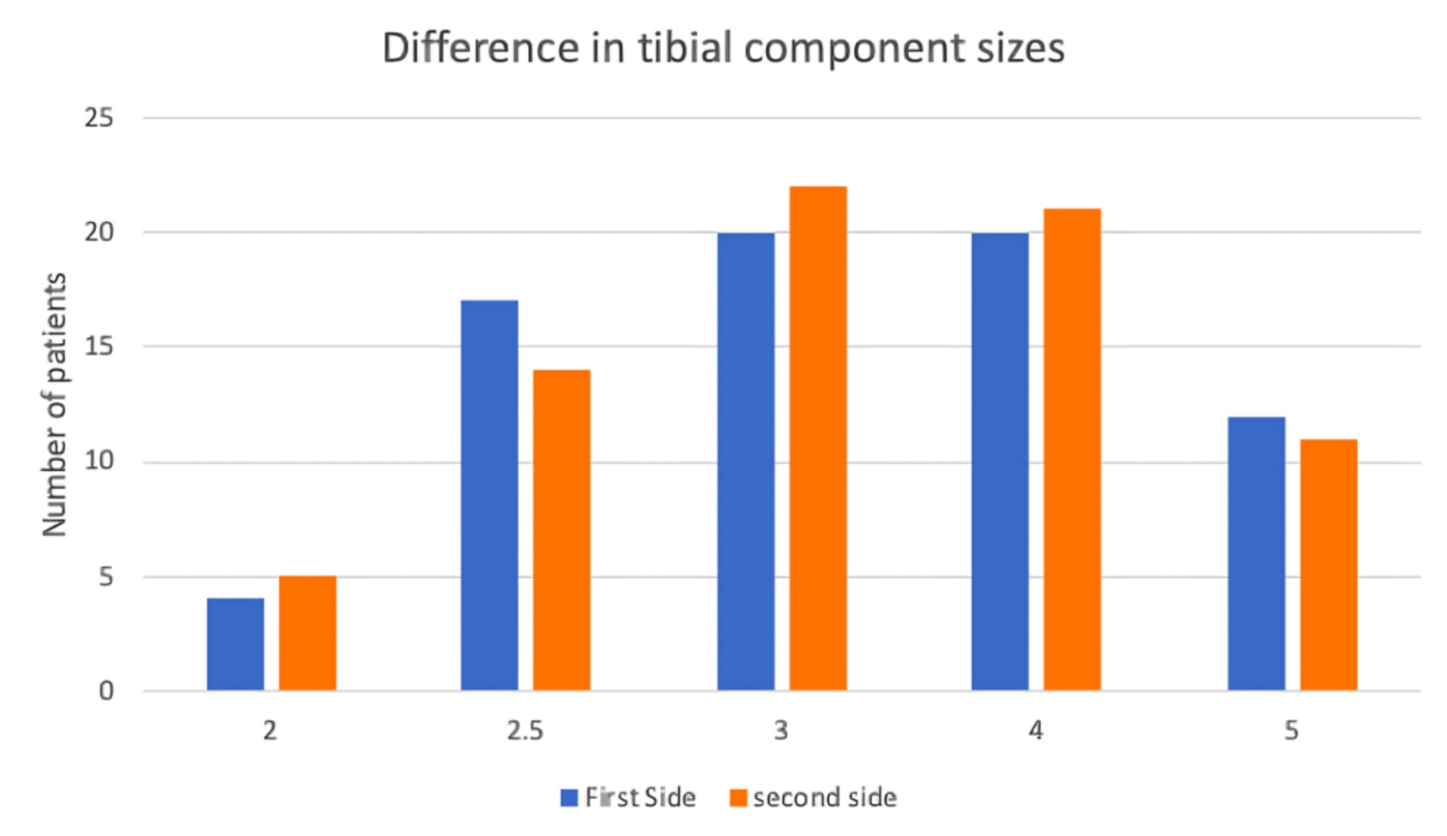
Tibial component sizes used in the cohort.

Most cases received a size 3 or 4 femoral component (Figure 2). Regarding the tibial component, 57/73 patients received implant sizes that were between 2.5 and 4 for first and second-side surgery (Figure 3).

Overall, 72/73 (98.6%) patients had both tibial and femoral components that were within one size of the first side operated on. Only one patient had an implant size difference of more than one, with the second side smaller in both components by two sizes. The gap between operations in this patient was 33 months, greater than the mean of the entire cohort. The mean interval between sides in the entire cohort was 28 months (range = 3-108 months). In the same patient, the BMI at the time of the first operation was 45 kg/m^2^. This decreased to 42 kg/m^2^ at the time of the second side operation; however, it was still greater than the average BMI of the entire cohort (35.5 kg/m^2^ at the time of the first operation; 35.7 kg/m^2^ at the time of the second). The implant used in this patient was sigma PFC cruciate retaining on both sides.

When comparing second-side surgery to first, the femoral component was of the same size in 54/73 patients. Only 12/73 patients had smaller femoral components implanted at the time of the second surgery, and 9/73 patients had bigger implants. Similar findings were noted regarding tibial component sizes. Overall, 47/73 patients had tibial components that were of the same size between first and second-side surgery. Further, 16/73 had a smaller tibial component implanted at the time of the second surgery and only 10 had a bigger component.

Total hip arthroplasty

**Figure 4:**
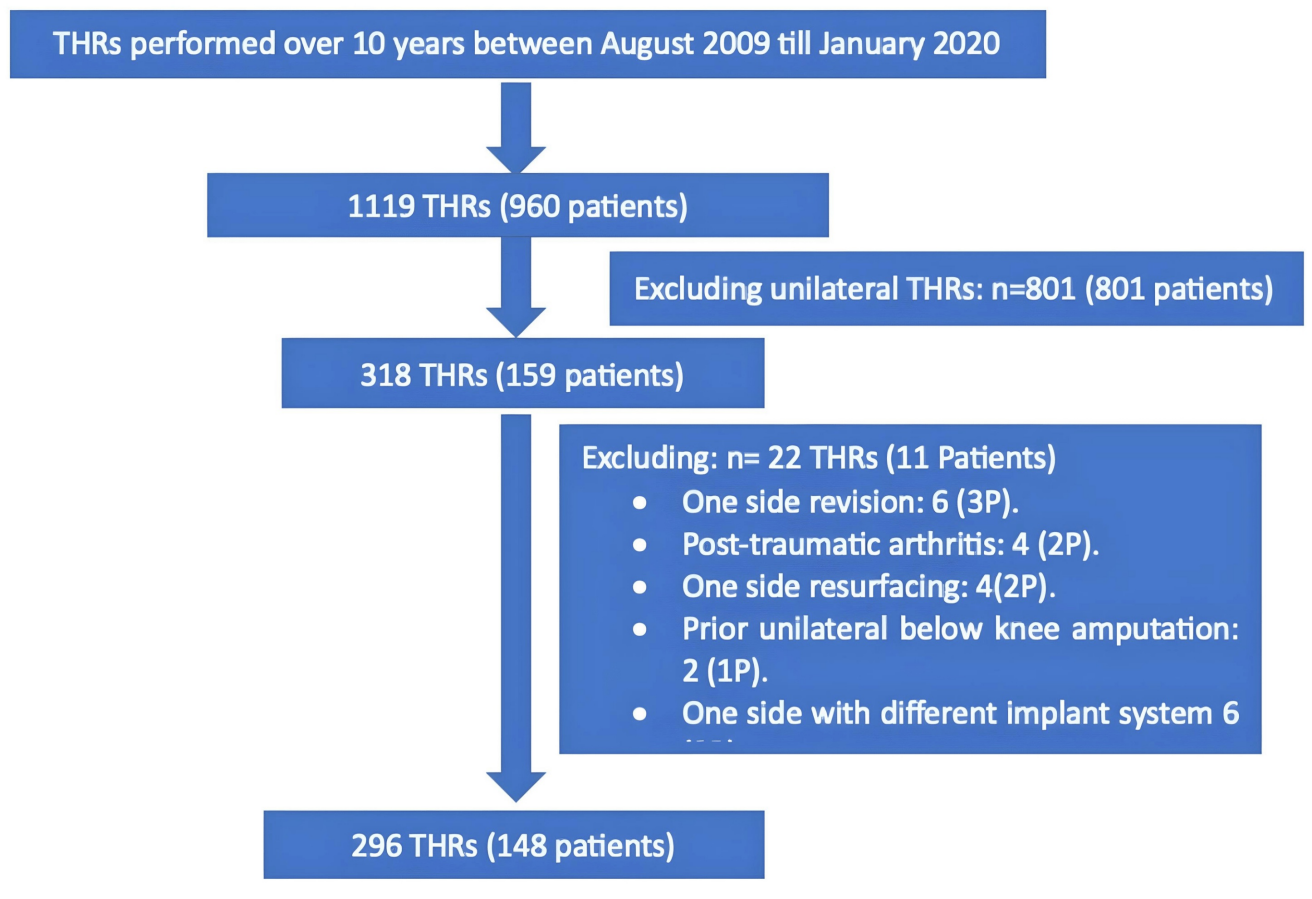
All total hip replacements (THRs) performed by the same surgeon over the defined study period. The selection of the study group and reasons for exclusion are presented.

For bilateral total hip replacements (BTHRs), 296 joints were operated on in 148 patients (Figure 4). Again, most patients (98/148) were older than 60 years of age, and the majority were female (93/148). Primary OA was evident on X-ray in 143/148 patients. Secondary bilateral OA due to adult dysplasia was seen in three patients and bilateral Perthes disease was evident in two patients (Table 2).

**Table 2:** Patient demographics including age, sex, and BMI (THR). BMI: body mass index; THR: total hip replacement; OA: osteoarthritis

Variables		n = 148
Sex	Male	55 (37.2%)
	Female	93 (62.8%)
Age at the time of the first side	<40	4 (2.7%)
	40–60	46 (31%)
	60–80	92 (62%)
	>80	6 (4%)
First side laterality	Right	74 (50%)
	Left	74 (50%)
BMI at the time of the first side	<25	23 (15.5%)
	25–30	40 (27%)
	30–40	68 (46%)
	>40	13 (8.7%)
	N/a	4 (2.7%)
Reasons for surgery	OA	143 (96.6%)
	Perthes	2 (1.35%)
	Adult dysplasia	3 (2%)

The mean interval between first and second-side surgery was 24 months (range = 2-102 months). Detailed implant sizes were available for all cases (296/296). All patients received a cementless Corail/Pinnacle system (DePuy Synthes) on both sides.

**Figure 5:**
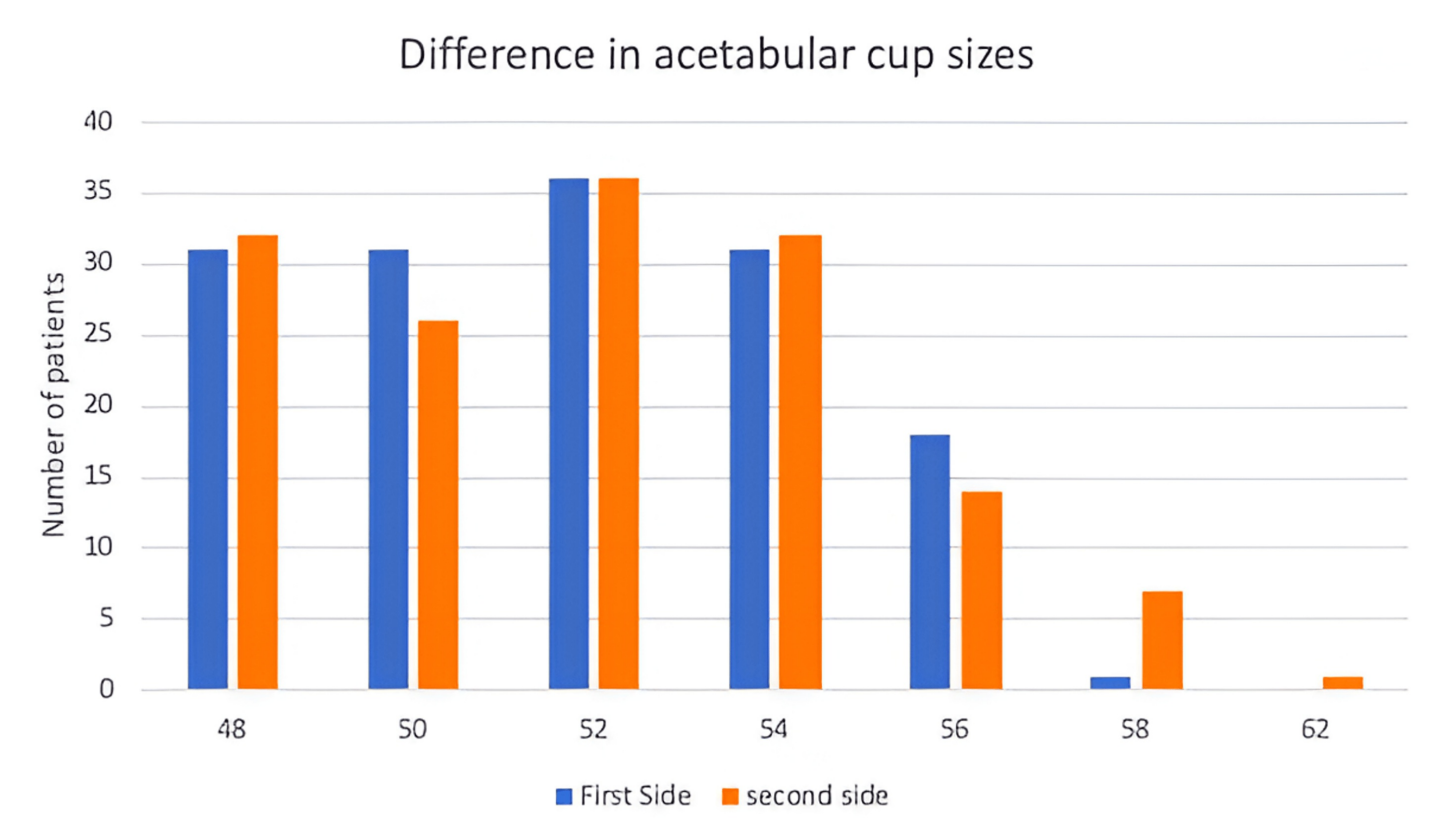
Differences in acetabular cup size between first and second-side surgery.

**Figure 6:**
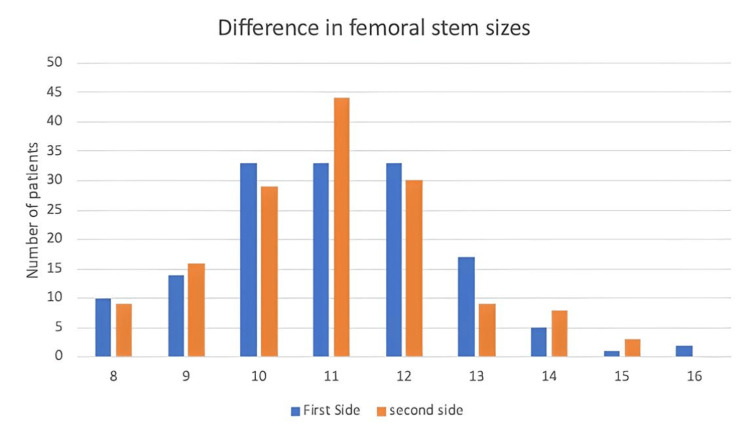
Differences in femoral stem sizes between first and second-side surgery.

Regarding the acetabular component, sizes varied between 48 and 52 in 98/148 patients for the first side operated on and 94/148 for the second side (Figure 5). Regarding the femoral component, sizes varied between 9 and 11 in 99/148 patients for the first side operated on and 103/148 patients for the second side (Figure 6).

Overall, 140/148 (94.6%) patients had an acetabular cup size that was within a one-size difference between first and second-side surgery. Regarding differences in femoral stem sizes, 130/148 (87.8%) had an implant that was again within a single size difference between first and second-side surgery.

Examining the 26 patients who had either an acetabular or femoral component that was not within a one-size difference between first and second-side surgery, the mean interval between sides was 28 months (range = 8-93). This, on average, is four months longer compared to the rest of the cohort, although this was not statistically significant (p = 0.609). The mean change in PROMs at one year for second-side surgery in the cohort of 26 was 26.59 (SD = 9.42), which is a better outcome compared to the first side operated on (mean change in PROMs of 25.48 (SD = 10.804) (p = 0.204). A comparison of this data with the entire cohort was not statistically significant. The mean change in PROMs following first-side surgery in the rest of the cohort was 26.34 (SD = 8.712) (p = 0.434), and 25.22 (SD = 10.064) (p = 0.727) following second-side surgery.

Further subgroup analysis regarding the interval between surgeries, gender, and mean change in Oxford Hip Scores (OHS) at the one-year follow-up showed no statistically significant differences (Tables 3, 4).

**Table 3:** Sub-analysis of differences in acetabular cup size according to time interval, gender, and BMI. BMI: body mass index; PROM: patient-reported outcome measure

	Whole cohort (n = 148)	Cup size within one of the first side operated on (n = 140)	Cup size within two of the first side operated on (n = 6)	Cup size within three of the first side operated on (n = 2)
Male	55	50	3	2
Female	93	90	3	0
Average BMI at first-side surgery	31.08	30.81	38	29
Average interval between first and second-side surgery (months)	24	25	23.40	50
Mean PROMs improvements between first and second-side surgery	25.22	25.15	26.33	26.50

**Table 4:** Sub-analysis of differences in femoral stem size according to time interval, gender, and BMI. BMI: body mass index; PROM: patient-reported outcome measure

	Whole group (n = 148)	Femoral stem size within one of the first side operated on (n = 130)	Femoral stem size within two of the first side operated on (n = 14)	Femoral stem size within three of the first side operated on (n = 4)
Male	55	48	6	1
Female	93	82	8	3
Average BMI at first-side surgery	31.08	31.20	28.85	30.75
Average interval between first and second-side surgery (months)	24	25.10	27.01	23.30
Mean PROMs improvements between first and second-side surgery	25.22	25.15	25	29.67

## Discussion

Following the COVID-19 pandemic and increased healthcare pressures, efforts to optimize healthcare processes are required to increase efficiency and productivity [2]. STR is one such process, aiming to utilize fewer instruments and trays, thereby improving efficiency, and decreasing the cost of sterilization and packing [10]. The principle involves a reduction of instruments per surgical tray and a reduction of surgical trays per surgery. Evidence is available confirming it to be cost-effective. In 2017, surgical teams in the United States applied STR in five surgical specialties and demonstrated a decrease in annual costs by up to an estimated $53,193 to $531,929 [10]. Similarly, in 2018, STR was applied specifically to orthopedic surgery, which led to a reduction in the total annual cost of $270,976 (20% overall cost reduction) [1].

In 2017, an orthopedic team from the United Kingdom applied the STR principle and managed to optimize surgical trays from four to two for a dynamic hip screw procedure; three to one for hip, knee, and shoulder arthroscopy; five to two for rotator cuff repair and shoulder stabilization; and three to one for total shoulder replacement and proximal humerus fracture fixation [9]. Based on local database figures for these procedures, the estimated number of used trays reduced from 2,785 to 1,015 (36.4%) per year [9]. Based on sterilization costs of £35 per tray, this is an annual saving of £61,950 [9].

This is the first study from the United Kingdom to explore the potential benefits and reliability of STR in elective bilateral staged THR and TKR. Our results show that the implant size used in the first-side surgery can be used as a reliable guide for the implant size used in the second. There is, therefore, potential to rationalize trays for the expected implant. Overall, 72/73 (98.6%) femoral and tibial TKR components were of the same or within one size of the first side operated on. Further, 140/148 (94.6%) THR acetabular components were of the same or within one size of the first side operated on, and 130/148 (87.8%) femoral stems were of the same or within one size of the first side operated on.

Although not 100% predictive, there is potential to reduce the number of required preparation devices, such as trials, broaches, and screw sets, and, in turn, reduce the number of trays used safely and effectively.

In our institution, surgical tray sterilization cost is calculated by the Central Sterile Services Department Unit (CSSD). For the TKR PFC Knee set, the total cost of sterilization is £226.76 per four-tray set. For the THR set, the total cost of sterilization is £215.36, also per four-tray set. The CSSD estimated the sterilization cost for our rationalized TKR tray to be £66.95 (a saving of £159.81), and for our rationalized THR tray, a cost of £64.10 (a saving of £151.26).

When considering 72/73 (98.6%) femoral and tibial TKR components were of the same or within one size of the first side operated on and identifying that the most common size of both tibial and femoral implants was size 3, the number of trays used can be reduced from four to one. This will lead to a reduction of cost by £159.81 per case.

Similarly, when considering 140/148 (94.6%) THR acetabular components and 130/148 (87.8%) femoral stems were of the same or within one size of the first side operated on and identifying that the most common size of the acetabular cup and femoral stem was 52 and 11, respectively, the number of trays used can be reduced from four to one. This will lead to a reduction of cost by £151.26 per case.

Using the formula, cost per case multiplied by the average number of expected yearly cases at our institution per surgeon, multiplied by the percentage of cases that had an implant within one size of the first side operated on, we were able to calculate the possible future savings on an annual basis. For TKRs (£159.81 x 26 x 98.63%), there is a possibility to save £4,098.14 per annum. For THRs, (£151.26 x 24 x97.29%) the potential savings would be £3,531.9. This is an average total saving of £7,630 per single arthroplasty surgeon at our institution.

The benefit of implant size awareness is not limited to STR. For example, the use of undersized implants is potentially harmful. In 2019, in Musgrove, Ireland, a cohort of 626 patients who received cementless Corail Pinnacle THRs were evaluated [11]. The study concluded that radiolucent lines would appear at zone 7 in the femoral component if it was undersized and recommended that careful preoperative templating should eliminate significant under-sizing [12]. The study also identified that when the Corail stem used was within one size of the template, there was a reduced chance of developing radiolucent lines in zone 7 [11].

In 2012, in Chicago, a new principle of template-directed instrumentation (TDI) was trialed that combined digital templating with limited intraoperative instruments [12]. Over a one-year period, 82 TKRs using TDI were reviewed. Preoperative templated component sizes were recorded using digital planning software that identified the two most likely sizes for tibial and femoral components [12]. This guided companies to prepare three lightweight instrument trays based on these sizes [12]. In 80/82 (97%) cases, the prepared sizes determined by TDI with three trays were sufficient. Preoperative templating predicted tibial component sizes in 90% and femoral component sizes in 83% of cases [12]. The average number of trays used with TDI was 3 compared to 7.5 in non-TDI TKAs [12]. With a standard sterilization and packaging fee of $26 per tray, using TDI saved $9,612 over the year. Therefore, digital templating and TDI proved to be a cost-effective method for performing primary TKR [12]. Similarly, a study published in 2021 demonstrated the predictive accuracy using a new technique of digitalized templating. A total of 141 consecutive TKAs in 113 patients were included. Overall, 164/282 (58%) TKA components were planned correctly and (274/282) 97% were within one size [13].

In 2018, a study conducted in Austria included 632 consecutive patients who underwent primary uncemented THA. Digital templating was done using the syngo-EndoMap software by Siemens Medical Solutions [14]. The implant size was predicted exactly in 264/632 (42%) for the femoral and in 231/632 (37%) for the acetabular component [14]. Overall, 547/632 (87%) of the femoral components and 494/632 (78%) of the acetabular cups were accurate within one size [14]. Our study demonstrated better results, with 140/148 (94.6%) and 130/148 (87.8%) of femoral and acetabular components, respectively, being within one size of the first side operated on.

Therefore, implant sizes can be highly anticipated. Used in conjunction with preoperative templating, the surgical team can confidently rationalize surgical trays. Additionally, any deviation from planned implant size should raise concerns and appropriate intraoperative checks for errors should be undertaken.

A limitation of this study is that we commented only on a single THR brand and two TKR brands. However, we believe these minimized possible differences among sides.

## Conclusions

This large cohort study confirms implant sizes used for first-side surgery are a reliable predictor for those required during second-side surgery in bilateral staged hip and knee replacement procedures. Most second-side implants were within one size of the implant utilized during first-side surgery for TKR and within two sizes for THRs. Used in conjunction with preoperative templating, surgical and procurement teams can confidently plan implant availability and rationalize surgical trays accordingly. This has the potential to improve theater efficiency and decrease sterilization costs.
